# Self-Consistent Study of GaAs/AlGaAs Quantum Wells with Modulated Doping

**DOI:** 10.3390/nano13050913

**Published:** 2023-03-01

**Authors:** John A. Gil-Corrales, Alvaro L. Morales, Carlos A. Duque

**Affiliations:** Grupo de Materia Condensada-UdeA, Instituto de Física, Facultad de Ciencias Exactas y Naturales, Universidad de Antioquia UdeA, Calle 70 No. 52-21, Medellín 50011, Colombia

**Keywords:** self-consistent calculation, quantum well, modulated doping, electromagnetic-induced transparency

## Abstract

In this work, the characterization and analysis of the physics of a GaAs quantum well with AlGaAs barriers were carried out, according to an interior doped layer. An analysis of the probability density, the energy spectrum, and the electronic density was performed using the self-consistent method to solve the Schrödinger, Poisson, and charge-neutrality equations. Based on the characterizations, the system response to geometric changes in the well width and to non-geometric changes, such as the position and with of the doped layer as well as the donor density, were reviewed. All second-order differential equations were solved using the finite difference method. Finally, with the obtained wave functions and energies, the optical absorption coefficient and the electromagnetically induced transparency between the first three confined states were calculated. The results showed the possibility of tuning the optical absorption coefficient and the electromagnetically induced transparency via changes to the system geometry and the doped-layer characteristics.

## 1. Introduction

Quantum wells (QWs) are semiconductor heterostructures that confine electrons (or holes) in one spatial dimension that are free in the other two dimensions. These systems are currently widely studied from both a theoretical and experimental point-of-view due to their multiple applications and the current possibility of generating very thin and high-quality layers, which was not possible twenty years ago when experimental techniques were not that advanced. In the case of a Al${}_{0.3}$Ga${}_{0.7}$As/GaAs/Al${}_{0.3}$Ga${}_{0.7}$As heterostructure, a quantum well was formed in the conduction band since the gap of Al${}_{0.3}$Ga${}_{0.7}$As was greater than the GaAs gap. An immediate consequence of this type of confinement was that the energy levels in the confinement direction became discrete and had a direct dependence on well width and donor density, among other parameters.

It is important to mention the many of the applications of QWs that include cutting-edge works in the optoelectronics field, such as the theoretical work developed in 2022 by Aissat et al. [1], in which multiple quantum wells (MQWs) were implanted inside the intrinsic region in a solar cell-type device based on InGaAsN/GaAs to improve efficiency by taking advantage of the absorption of low-energy photons. In this work, Aissat et al. obtained a theoretical external quantum efficiency (EQE) greater than 80%. In this same optoelectronics field, Roy et al. [2] analyzed the effect of dark currents from a theoretical perspective for the development of a CdS/ZnSe photodetector for mid-infrared based on an array of MQWs; their results showed the high detection capabilities of this system, as compared to other materials and different compositions. In 2021, Yu and Dang [3] synthesized colloidal metal chalcogenide quantum wells (CQWs) in an experimental field for laser applications ten years after the successful synthesis of two-dimensional colloidal systems; in this work, the authors categorized different systems according to the confinement of photons and laser mechanisms, such as amplification of spontaneous emission, a laser with a cavity, and a multi-photon-pumped laser.

It is worth mentioning some applications in more diverse fields, such as the work of Hu and Zhang [4], who in 2020 proposed a ZnO QWs topological insulator piezoelectric device. They found that under stress, a piezoelectric field was induced that caused the QWs to behave as topological insulators; this behavior depended, to a large extent, on the QW’s width. Regarding the application of QW in electronics in more recent works, in 2022, Zhou et al. [5] used machine-learning methods to demonstrate that through the application of neural networks, it was possible to solve the wavelength of intersubband transitions in piezo-phototronic GaN/AlN transistors since their hidden layers could be accurately approximated by any continuous function. In 2022, in an interesting experimental work by Park et al. [6], they studied the effective mobility of InGaAs/InAlAs QW for direct application in high electron mobility transistors (HEMTs) on an InP substrate. They achieved a significant reduction in the gate leakage current, which obtained precise measures of effective mobility.

In the applications mentioned above, QW and MQW systems using various materials were reported. Systems based on GaAs/AlGaAs were particularly interesting since they have been widely studied materials. Due to their particular characteristics of tuning electronic properties with external parameters, they have been of great use for multiple applications in various fields. In 2023, Turkoglu et al. [7] investigated the photoluminescence of GaAs/AlGaAs MQWs grown by metal–organic vapor-phase epitaxy (MOVPE); they analyzed the transition between the bands in the structure and their changes under different temperatures and external electric fields. In 2022, Makhov et al. [8] experimentally investigated the effect of the current drag of photons in the mid-infrared range, as it corresponded to the intersubband optical electron transitions in GaAs/AlGaAs QWs at room temperature; they studied the dependence of the change in the refractive index in the lateral current for different polarizations.

Currently, this type of material is of great interest since it enables optoelectronic applications at terahertz frequencies. Al-Naghmaish et al. [9] studied the optical response of a system of QWs as photodetectors under the effect of magnetic and electric fields and intense laser fields. Their work demonstrated the possibility of adjusting and tuning the absorption coefficient and the refractive index for terahertz applications. The recent advances in these materials have not been found exclusively from the theoretical perspective. It is necessary to highlight the advances in experimental techniques that have allowed a better characterization of these heterostructures [10,11]. For example, in 2021, Zhang et al. [12] performed the insertion of GaAs layers to improve the properties of InGaAs/AlGaAs MQWs grown by metal–organic chemical vapor deposition. The study showed that when the GaAs layer was approximately 6 nm, the maximum properties of the system were revealed.

When characterizing a QW system, either for a possible application in an optoelectronic device or as an active element in an electrical circuit, it is necessary to consider materials that include doping since, when doping the material, the electronic transport properties are amplified as are experimentally measurable characteristics, such as electric current, conductance, etc. Therefore, to obtain a better fit between the theoretical and experimental results, it has been necessary to consider QW models that consider the electrostatic potential generated by a donor density. In 2023, Dakhlaoui et al. [13] numerically studied the optical response in Manning-like GaAs/AlGaAs double QWs, including the effects of doped impurities. They added an *n*-doped layer in two different positions of the potential. Among the reported results, there was evidence of a loss of degeneracy of the energy levels depending on the position of the doped layer and the density of the donors; this feature significantly modified the optical properties of the system. In 2021, Sadonov et al. [14] investigated, both theoretically and experimentally, the dependence of the electron transport properties of a two-dimensional electron gas on sheet-doping concentrations in one-sided δ-doped pseudo-morphic AlGaAs/InGaAs/GaAs QWs. Among the results of their report, the dependence of the transport relaxation times with $n_{H}$ (ionized donors concentration) exhibited a non-monotonic behavior due to the competition of the Fermi momentum increase and the scattering angle due to the $n_{H}$ variation.

These heterostructures are widely characterized via their optical properties, such as through linear and nonlinear optical absorption coefficients; electromagnetically induced transparency (EIT); and changes in refractive index, among others. This has been evidenced by recent works, such as that of Rodríguez-Magdaleno et al. [15], in which a theoretical study of the electronic structure and the intersubband-related optical absorption coefficient for symmetrical double-delta-doped GaAs QW was carried out. Among the reported results, the modification of the absorption peak position due to the presence of donor impurity atom was noted. In another interesting theoretical work, Jayarubi et al. [16] calculated the nonlinear optical absorption coefficient and the EIT in GaAs/InAs/GaAs QWs. The optical susceptibilities, the detuning parameters, and the Rabi frequency were also analyzed. In general, it was a complete work from a theoretical perspective for understanding the effects of EIT on low-dimensional heterostructures.

Obviously, a system of QWs composed of GaAs/AlGaAs with internal doping remains a challenge in current development, so this work characterized a GaAs QW with AlGaAs barriers with a modification in the bottom of the conduction band due to the electrostatic potential generated by a δ-doped layer. The study considered variations in the energy spectrum, probability density, electron density, and Fermi level. In this work, geometric modifications, such as the QW width, and non-geometric modifications, such as the doped region width and the doped layer position, were carried out.

Due to the significant growth of experimental techniques in recent years, it is currently possible to grow very thin doped regions (delta type) of very precise widths and of high quality inside heterostructures, such as QWs, of various materials, as evidenced by works such as that of F. Ishikawa et al. [17], who studied the energy-band engineering with nitrogen δ-doping in GaAs-related quantum structures.In the same way, by means of techniques such as molecular beam epitaxy, it is possible to control the position of doped layers, a reference to the application of said method was presented in a report by H. Khmissi et al. [18]. In 2018, S. Kang et al. [19] used an Si δ-doping technique to fabricate high-performance GaAs tunnel diodes (TDs).

By including a doped region inside a heterostructure, some of its properties can be improved; for example, the cathodoluminescence (CL) in InP quantum dots grown on an InAlP matrix was improved by approximately 16-times through the modulation of the position of a silicon-doped delta layer. This was shown in an experimental work by X. B. Zhang et al. [20]. In another interesting report, X. Chen et al. [21] performed a characterization of a GaAs-based high-speed and high-sensitivity delta-doped resonant cavity-enhanced HMSM photodetector, identifying that the growth of a doped delta layer inside the heterostructure improved the photocurrent spectral response, the dark current, the time response, and the capacitance–voltage measurements, as compared to un-doped systems. Finally, V. V. Vainberg et al. [22] experimentally modulated the position of the delta layer inside and outside of a GaAs/InGaAs/GaAs QW, and they identified a significant increase in electron mobility when the delta layer was located in barrier regions, as compared to the measurements of the delta layer inside the QW.

As an application of our findings (eigenvalues and eigenfunctions), the linear absorption coefficient and the EIT were calculated. The self-consistent method, combined with the finite difference method (FDM), was used to solve the coupled Schrödinger, Poisson, and charge-neutrality equations. The paper is organized as follows: In Section 2, we present our theoretical model; Section 3 is devoted to the results and their corresponding discussions; finally, in Section 4, the conclusions are given.

## 2. Theoretical Model

**Figure 1:**
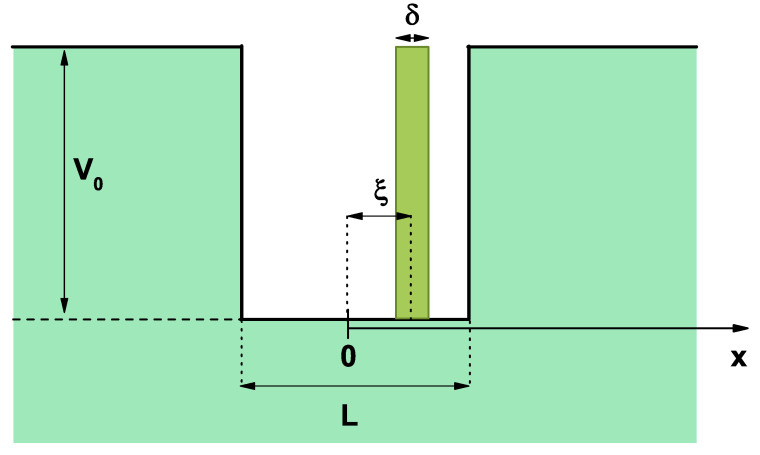
Schematic view corresponding to a quantum well of height $V_{0}$ and width *L* at the center of the *x*-axis origin. The doped delta layer of δ width located at a ξ distance from the origin of the coordinates is shown in dark green.

The system to be studied corresponded to a GaAs QW with Al${}_{0.3}$Ga${}_{0.7}$As barriers, as shown in Figure 1. This was characterized by a a height $V_{0}$ and width *L* as the center of the *x*-axis origin.The *L*-parameter was the well-width at the center of the *x*-axis origin. The system presents an *n*-type doped layer, located at a distance of ξ from the origin and of a width δ, represented by a green rectangle.

The calculations presented were assessed at room temperature, as one of the main objectives of the work was to understand the physics of this type of heterostructure, and as a result, our findings could be implemented more directly in electronic devices or in widespread applications. (It is more difficult to identify applications for devices that only function at low temperatures.) The effect of operating at lower temperatures in this type of system has not been explored by this model; however, a decrease in the occupancy of the confined states could be expected as a direct effect of the decrease in temperature, due to a proportional self-consistent Fermi level. As a consequence, the density of electrons occupying discrete states could decrease, which could impair the characterization of the system by optics, since the number of charge carriers available to perform transitions would also decrease.

At room temperature, it was reasonable to consider a total ionization of the donor atoms, because for the implementation of this type of heterostructure in practical applications or in devices, it would be necessary to have a set of half-occupied states that increased electronic mobility through the system, that is, to have a certain electronic occupancy in the conduction band. Although complete ionization has been assumed in this work, it was evident that the percentage of the ionized atoms was dependent on the system temperature, in addition to the atom donor density [23]. In order for theoretical calculations to be useful for potential applications, we conducted this experiment at room temperature, as for GaAs at this temperature, it was possible to consider a “relatively high ionization”, even accounting for the increase in impurity ionization energy for the case of one-dimensional confinement. In [13,24,25], they presented various QWs of different materials in which total ionization with different densities at room temperature were considered, and they reported very interesting results.

To determine the electrostatic potential of the interaction between electrons and ions, Poisson’s equation was calculated with the corresponding charge density. This charge density generated a Hartree potential that modified the bottom profile of the conduction band to identify the correct potential, states, energies, etc. The problem was approached using the self-consistent formalism, which is detailed in the next section.

### 2.1. Self-Consistent Method

The self-consistent method [25,26,27] is a particularly useful method for calculating wave functions, energy levels, charge densities, electron densities, and Fermi levels in systems that have regions with a certain donor density (acceptor density), that is, doped systems. This method considers the disturbance in the system generated by the electrostatic potential due to the donor’s density, and this term must be included in the Hamiltonian at a low percentage since if 100% of this disturbance was included, the convergence would likely not be achieved.

According to Figure 1, we defined a confinement potential that accounted for the magnitude of the confinement potentials and the sizes of the structure. We defined the electron confinement potential as follows:

(1) $$V_{c}\left(x\right)\equiv \left\{\begin{array}{cc} V_{0}\;, & \mathrm{if}\;|x|>L/2\\ 0\;, & \mathrm{if}\;|x|\leq L/2\;. \end{array}\right.$$

The starting point of the self-consistent method was to solve the time-independent Schrödinger equation, considering only the electronic confinement potential, that is, the term $V^{\left(0\right)}\left(x\right)\equiv V_{c}\left(x\right)$,

(2) $$-\frac{\hbar^{2}}{2\;m^{*}}\frac{\partial^{2}\psi_{j}^{\left(0\right)}\left(x\right)}{\partial x^{2}}+V^{\left(0\right)}\left(x\right)\psi_{j}^{\left(0\right)}\left(x\right)=E_{j}^{\left(0\right)}\psi_{j}^{\left(0\right)}\left(x\right)\;.$$

In this equation, *ℏ* corresponds to the reduced Planck constant, $m^{*}$ is the effective mass that is taken equal in the region of wells and barriers, and $\psi_{j}^{\left(0\right)}\left(x\right)$ is the wave function of the system corresponding to the eigenvalue $E_{j}^{\left(0\right)}$ (*j* corresponds to the *j*-th state, and the subscript (0) indicates the initial step in the self-consistent method).

Equation (2) could be solved using any numerical method; in particular, in this work, FDM [28,29] was used to solve all the equations involved in the problem (this method is explained in the next section). Once Equation (2) was solved, a set of eigenfunctions and their corresponding eigenvalues, $\left\{\psi_{j}^{\left(0\right)}\left(x\right),E_{j}^{\left(0\right)}\right\}$, were obtained. However, the delta layer had to contain a volumetric donor density, $n_{d}$ (electrons per cubic meter); additionally, the complete system must comply with the charge-neutrality condition, which posits that the total number of electrons must be equal to the number of ionized donors per unit area, that is, the following relationship must be met:

(3) $$n_{d}\;\delta =\sum_{j}\frac{m^{*}\;k_{B}\;T}{\pi \;\hbar^{2}}\log\left[1+\exp\left(\frac{E_{f}^{\left(0\right)}-E_{j}^{\left(0\right)}}{k_{B}\;T}\right)\right]\;,$$

where δ is the width of the delta doped layer, $k_{B}$ is the Boltzmann constant, *T* is the system temperature (300 K in this work), $E_{f}^{\left(0\right)}$ is the Fermi level, and $E_{j}^{\left(0\right)}$ are the eigenvalues obtained by means of Equation (2). In Equation (3), the only unknown term was the Fermi level, $E_{f}^{\left(0\right)}$. Clearly, this expression corresponded to a transcendental equation that could be solved for $E_{f}^{\left(0\right)}$. Based on the above, it followed that the second step of the self-consistent procedure was to use the charge-neutrality relation to calculate the Fermi level.

Once the wave functions were calculated, the corresponding eigenvalues and the Fermi level in the system were obtained; based on these, an expression for the electron density associated with the occupation of each of the states was obtained, as follows:

(4) $$n^{\left(0\right)}\left(x\right)=\;\sum_{j}\frac{m^{*}\;k_{B}\;T}{\pi \;\hbar^{2}}\log\left[1+\exp\left(\frac{E_{f}^{\left(0\right)}-E_{j}^{\left(0\right)}}{k_{B}\;T}\right)\right]{\left|\;\psi_{j}^{\left(0\right)}\left(x\right)\right|}^{2}\;.$$

The next step was to calculate the electrostatic potential, and this procedure was carried out by solving Poisson’s equation, considering a charge density associated with the ionized donors and the electron density:

(5) $$\frac{d^{2}V_{H}^{\left(0\right)}\left(x\right)}{dx^{2}}=\frac{e^{2}}{\epsilon \;\epsilon_{0}}\left(n_{d}\left(x\right)-n^{\left(0\right)}\left(x\right)\right)\;,$$

where $V_{H}^{\left(0\right)}\left(x\right)$ is the Hartree potential obtained in the first self-consistency step, *e* is the electron charge, $\epsilon_{0}$ is the vacuum dielectric permittivity, and ϵ is the material’s relative permittivity (which was assumed to be constant in the whole heterostructure). Note that in Equation (5), full ionization was being considered; this was a reasonable assumption for this material at room temperature. The function $n_{d}\left(x\right)$ used the value of $n_{d}$ in the region where the doped delta layer was located and was equal to zero at the other *x*-points. From the above, we defined a new electronic potential starting from the initial potential $V^{\left(0\right)}\left(x\right)=V_{c}\left(x\right)$, in the form $V^{\left(1\right)}\left(x\right)=95\%V^{\left(0\right)}\left(x\right)+5\%\left[V_{c}\left(x\right)-V_{H}^{\left(0\right)}\left(x\right)\right]$, that is, 5% of the Hartree disturbance potential had been included (the 5% value was chosen to guarantee that a significantly large perturbation in the electronic confinement potential that could lead to a numerical divergence was not generated. The chosen value, despite increasing the computational time, guaranteed a correct convergence). With this potential, we solved the Schrödinger equation again, as follows:

(6) $$-\frac{\hbar^{2}}{2\;m^{*}}\frac{\partial^{2}\psi_{j}^{\left(1\right)}\left(x\right)}{\partial x^{2}}+V^{\left(1\right)}\left(x\right)\psi_{j}^{\left(1\right)}\left(x\right)=E_{j}^{\left(1\right)}\psi_{j}^{\left(1\right)}\left(x\right)\;.$$

Through the solution of Equation (6), a new set of eigenfunctions and eigenvalues $\left\{\psi_{j}^{\left(1\right)}\left(x\right),E_{j}^{\left(1\right)}\right\}$ was obtained in order to repeat the procedure again. In general, in an *m*-th step, it was necessary to solve the Schrödinger equation:

(7) $$-\frac{\hbar^{2}}{2\;m^{*}}\frac{\partial^{2}\psi_{j}^{\left(m\right)}\left(x\right)}{\partial x^{2}}+V^{\left(m\right)}\left(x\right)\psi_{j}^{\left(m\right)}\left(x\right)=E_{j}^{\left(m\right)}\psi_{j}^{\left(m\right)}\left(x\right)\;.$$

In this equation, the self-consistent potential had the form, as follows:

(8) $$V^{\left(m\right)}\left(x\right)=95\%V^{\left(m-1\right)}\left(x\right)+5\%\left[V_{c}\left(x\right)-V_{H}^{\left(m-1\right)}\left(x\right)\right]\;,$$

where the Hartree potential $V_{H}^{\left(m-1\right)}\left(x\right)$ is obtained by solving the equation:

(9) $$\frac{d^{2}V_{H}^{\left(m-1\right)}\left(x\right)}{dx^{2}}=\frac{e^{2}}{\epsilon \;\epsilon_{0}}\left(n_{d}\left(x\right)-n^{\left(m-1\right)}\left(x\right)\right)\;,$$

with

(10) $$n^{\left(m-1\right)}\left(x\right)=\;\sum_{j}\frac{m^{*}\;k_{B}\;T}{\pi \;\hbar^{2}}\log\left[1+\exp\left(\frac{E_{f}^{\left(m-1\right)}-E_{j}^{\left(m-1\right)}}{k_{B}\;T}\right)\right]{\left|\;\psi_{j}^{\left(m-1\right)}\left(x\right)\right|}^{2}\;.$$

In this equation, the Fermi level associated with the m−1 self-consistent step was calculated again using the charge neutrality condition:

(11) $$n_{d}\;\delta =\sum_{j}\frac{m^{*}\;k_{B}\;T}{\pi \;\hbar^{2}}\log\left[1+\exp\left(\frac{E_{f}^{\left(m-1\right)}-E_{j}^{\left(m-1\right)}}{k_{B}\;T}\right)\right]\;.$$

The parameter used to verify the convergence was the Fermi level $E_{f}$ (in a completely analogous way, the wave function, the energies, etc. could be used). In this way, in each cycle of the procedure, the quantity $|E_{f}^{\left(m\right)}-E_{f}^{\left(m-1\right)}|$ was calculated. If in an *N*-th step, this expression was less than a certain tolerance (for this work, a value of $10^{-5}$ eV was sufficient), then the self-consistent process stopped, and the set of solutions found in this last step $\left\{\psi_{j}^{\left(N\right)}\left(x\right),V^{\left(N\right)}\left(x\right),E_{j}^{\left(N\right)},E_{f}^{\left(N\right)},V_{H}^{\left(N\right)}\left(x\right),n^{\left(N\right)}\left(x\right)\right\}$ must correspond to the self-consistent solution of the problem.

**Figure 2:**
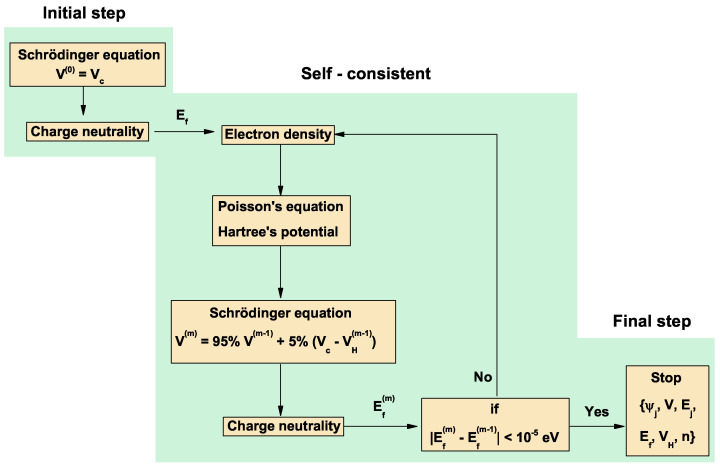
Block diagram corresponding to the self-consistent algorithm.

Figure 2 presents a block diagram that summarizes the self-consistent procedure as follows: starting from the solution of the Schrödinger equation with the potential $V^{\left(0\right)}\left(x\right)\equiv V_{c}\left(x\right)$; from the calculated eigenstates and eigenvalues, the charge neutrality was used to determine the Fermi level. With this Fermi level and the eigenstates, the electron density was constructed; from this density and considering the charge contribution generated by the doped delta layer, a charge density was defined that was entered into Poisson’s equation to obtain the Hartree potential. With this Hartree potential, a new electronic potential was built, and the Schrödinger equation was solved again with this new potential. At this point, a new set of eigenstates and eigenvalues was obtained that were used again to calculate a new Fermi level through charge neutrality. Finally, the Fermi level was compared, in this step, with that obtained in the previous step. If a value less than a certain previously defined tolerance was obtained, the procedure stopped; if, on the contrary, a value greater than the tolerance was obtained, we repeated the process until convergence was obtained.

### 2.2. Finite Difference Method (FDM)

**Figure 3:**
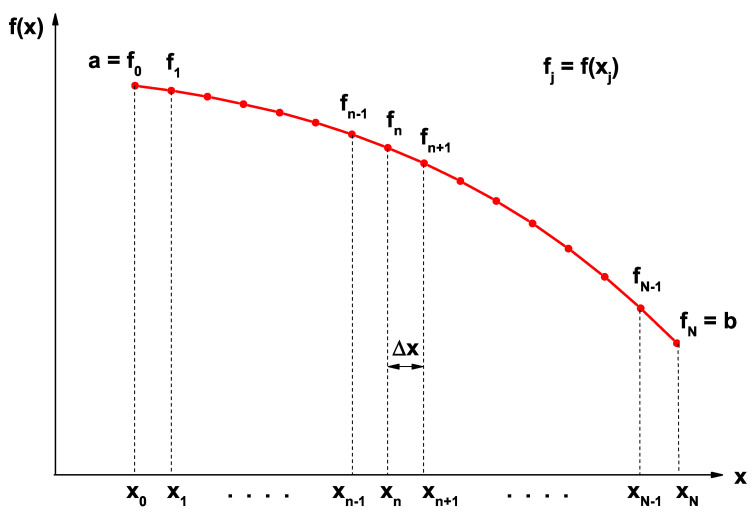
Representative schematic of a one-dimensional point mesh used to develop the finite difference method.

The FDM is widely used for solving differential equations. The advantage of implementing this method was to convert the differential equation into a set of algebraic equations that could be solved by any matrix or diagonalization method. Figure 3 represents a scheme in which the function f(x) (red curve) had been discretized in a set of points according to equidistant grid N+1 points on the *x*-axis, in such a form that was fulfilled by definition: $f_{j}\equiv f\left(x_{j}\right)$ for all $x_{j}$ in the interval $\left(x_{0},x_{N}\right)$.

From the above, it clearly follows that each point $x_{j}$ can be represented as $x_{j}=x_{0}+j\Delta x$. The starting point for the development of the method consisted of the ordinary definition of the first-order derivative at a $x_{k}$ point using central differences: $df\left(x_{k}\right)/dx=\lim_{\Delta x\to 0}\left[f\left(x_{k}+\Delta x\right)-f\left(x_{k}-\Delta x\right)\right]/2\Delta x$. In the limit in which Δx<<1, we approximated the derivative at a $x_{k}$ point, as follows:

(12) $$\frac{df(x_{k})}{dx}\approx \frac{f\left(x_{k+1}\right)-f\left(x_{k-1}\right)}{2\Delta x}=\frac{f_{k+1}-f_{k-1}}{2\Delta x}\;.$$

To ensure good precision in the results, it was necessary that the parameter Δx be much smaller than the size of the interval corresponding to the domain of *x*, that is, it must be true that $\Delta x<<x_{N}-x_{0}$. According to Equation (12), this was a well-known expression to represent the first derivative of a function at the point $x_{k}$. Therefore, we could start from the derivative expression using forward differences or backward differences to obtain the second derivative of a function f(x) at a point $x_{k}$ through central differences, that is:

(13) $$\frac{d^{2}f\left(x_{k}\right)}{dx^{2}}\approx \frac{\frac{f_{k+1}-f_{k}}{\Delta x}-\frac{f_{k}-f_{k-1}}{\Delta x}}{\Delta x}=\frac{f_{k-1}-2f_{k}+f_{k+1}}{\Delta x^{2}}\;.$$

Furthermore, an expression was found to calculate the second derivative of a function f(x) at the point $x_{k}$ by means of central differences. Note that in Equations (12) and (13), *k* had integer values only in the interval (1,N−1). The terms $f_{0}$ and $f_{N}$ corresponded to boundary conditions (see Figure 3) for the functions with values *a* and *b* that were assumed to be known: $f_{0}=f\left(x_{0}\right)=a$ and $f_{N}=f\left(x_{N}\right)=b$, respectively. Evaluating Equations (12) and (13) for some *k* values, it was easy to show that the first and second derivatives of a function on the given interval could be written in matrix form, as follows:

(14) $$\frac{df(x)}{dx}=\;\frac{1}{2\Delta x}\left(\begin{array}{cccccccccc} 1 & 0 & 0 & 0 & 0 & 0 & \cdots & 0 & 0 & 0\\ -1 & 0 & 1 & 0 & 0 & 0 & \cdots & 0 & 0 & 0\\ 0 & -1 & 0 & 1 & 0 & 0 & \cdots & 0 & 0 & 0\\ 0 & 0 & -1 & 0 & 1 & 0 & \cdots & 0 & 0 & 0\\ \vdots & \; & \vdots & \; & \ddots & \; & \vdots & \; & \vdots & \\ 0 & 0 & 0 & 0 & 0 & \cdots & 0 & -1 & 0 & 1\\ 0 & 0 & 0 & 0 & 0 & 0 & \cdots & 0 & 0 & 1 \end{array}\right)\left(\begin{array}{c} f_{0}\\ f_{1}\\ f_{2}\\ f_{3}\\ \vdots \\ f_{N-1}\\ f_{N} \end{array}\right)$$

and

(15) $$\frac{d^{2}f\left(x\right)}{dx^{2}}=\;\frac{1}{\Delta x^{2}}\left(\begin{array}{cccccccccc} 1 & 0 & 0 & 0 & 0 & 0 & \cdots & 0 & 0 & 0\\ 1 & -2 & 1 & 0 & 0 & 0 & \cdots & 0 & 0 & 0\\ 0 & 1 & -2 & 1 & 0 & 0 & \cdots & 0 & 0 & 0\\ 0 & 0 & 1 & -2 & 1 & 0 & \cdots & 0 & 0 & 0\\ \vdots & \; & \vdots & \; & \ddots & \; & \vdots & \; & \vdots & \\ 0 & 0 & 0 & 0 & 0 & \cdots & 0 & 1 & -2 & 1\\ 0 & 0 & 0 & 0 & 0 & 0 & \cdots & 0 & 0 & 1 \end{array}\right)\left(\begin{array}{c} f_{0}\\ f_{1}\\ f_{2}\\ f_{3}\\ \vdots \\ f_{N-1}\\ f_{N} \end{array}\right)\;.$$

Equations (14) and (15) were the matrix representation of the first and second derivatives, respectively, of a function f(x) on the interval $\left(x_{0},x_{N}\right)$ with boundary conditions $f(x_{0})=a$ and $f(x_{N})=b$. By means of this last representation of the second derivative, we needed to solve Equations (2) and (5) at each self-consistent step.

### 2.3. Absorption Coefficient and Electromagnetically Induced Transparency (EIT)

**Figure 4:**
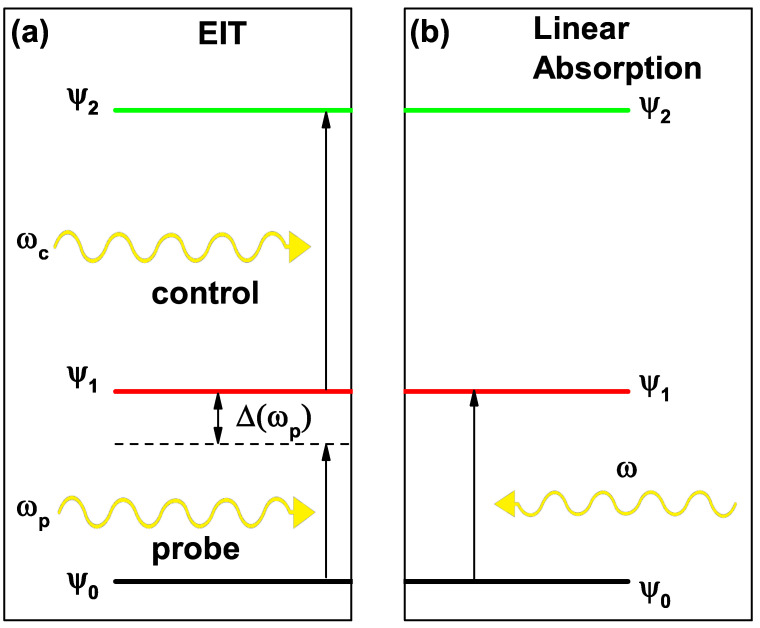
(**a**) diagram of a three-level system ($\Psi_{0},\Psi_{1},\Psi_{2}$) in a cascade arrangement, interacting with two electromagnetic fields of frequencies $\omega_{c}$ and $\omega_{p}$ (control and probe, respectively). (**b**) diagram of the absorption of a photon of frequency ω between states $\Psi_{0}$ and $\Psi_{1}$.

Once the wave functions and the energies corresponding to each state had been calculated in a self-consistent manner, it was possible to calculate the absorption coefficient (in linear order for this case) using the traditional relationship that was obtained by an expansion of the density matrix and the Von Neumann equation. This effect corresponded to the photon absorption of energy ℏω with a higher probability in the energy corresponding to the transition energy between states $E_{fi}-E_{in}$ (in this work, it had been calculated with $E_{in}=E_{0}$ and $E_{fi}=E_{1}$, see Figure 4b) [30,31,32]:

(16) $$\alpha \left(\omega \right)=\sqrt{\frac{\mu }{\epsilon \;\epsilon_{0}}}\times \frac{|M_{in-fi}{|}^{2}\;{\tilde{\sigma }}_{in-fi}\;\hbar \;\omega /\tau_{in}}{{\left(\Delta E-\hbar \;\omega \right)}^{2}+{\left(\hbar /\tau_{in}\right)}^{2}}\;,$$

where for *x*-polarized incident radiation, $|M_{in-fi}|\equiv |\langle \Psi_{in}\left|e\;x\right|\Psi_{fi}\rangle |$ is the dipole matrix element, $\Delta E=E_{fi}-E_{in}$, $\tau_{in}$ is the intersubband relaxation time, and

(17) $${\tilde{\sigma }}_{in-fi}=\frac{m^{*}\;k_{B}\;T}{L\;\pi \;\hbar^{2}}\times \ln\left(\frac{1+\exp\left[\left(E_{f}-E_{in}\right)/k_{B}T\right]}{1+\exp\left[\left(E_{f}-E_{fi}\right)/k_{B}T\right]}\right)\;.$$

The previous Equation (17) considered the electron state occupations according to the Fermi level and temperature.

However, the EIT was an effect in which a material that presented a high absorption at a certain wavelength between two states ($\Psi_{0}$ and $\Psi_{1}$) became transparent for that wavelength by taking advantage of the system interaction with two electromagnetic fields that had been coupled to three levels in the system. These fields were normally known as a probe field with a frequency of $\omega_{p}$ and a control field with a frequency of $\omega_{c}$ [33,34]. The probe field was associated with the coupling of the photons to the $\Psi_{0}\to \Psi_{1}$ electronic transition. By considering only the probe field, it generated an absorption peak located near the $E_{1}-E_{0}$ energy transition. However, the control field referred to the coupling of the photons to the transition $\Psi_{1}\to \Psi_{2}$. Depending on the characteristics of the control field, a destructive interference effect could be generated to attenuate the peak of maximum absorption mentioned above, making the system transparent for the energy in which it presented its maximum absorption. Figure 4a shows the scheme of this system.

For the phenomenon of EIT to occur, a three-level system ($\Psi_{0},\Psi_{1},\Psi_{2}$) was required with dipole coupling between $\Psi_{0},\Psi_{1}$ and $\Psi_{1},\Psi_{2}$ and forbidden coupling (or much smaller than those previously mentioned) between $\Psi_{0},\Psi_{2}$ (cascade arrangement). In addition, through the application of two electromagnetic fields (probe and control fields), a destructive quantum interference effect was generated between the two electronic transitions, resulting in the cancellation of the optical absorption, even in the presence of the applied field [35].

For absorption cancellation to occur, the system must initially be prepared with an electronic occupancy in the ground state, and thus, the imaginary part of the susceptibility (at resonance) was proportional to the atomic decay between $\Psi_{0}$ and $\Psi_{2}$ (which was much less than 1 since it was a prohibited coupling). Since the imaginary part of the susceptibility was proportional to the optical absorption, this was attenuated, resulting in the EIT effect.

We considered a three-level system $\Psi_{0},\Psi_{1}$, and $\Psi_{2}$, with corresponding energies $E_{0},E_{1}$, and $E_{2}$, respectively. When a control field with frequency $\omega_{c}$ was coupled to states $\Psi_{1}\to \Psi_{2}$, this field could modify the susceptibility of a probe field with the frequency $\omega_{p}$ associated with the $\Psi_{0}\to \Psi_{1}$ transition. The Hamiltonian that described this situation corresponded to a sum of the unperturbed Hamiltonian ${\hat{H}}_{0}$ (not including electromagnetic fields), plus the radiation-matter interaction part ${\hat{H}}^{*}$:

(18) $$\hat{H}={\hat{H}}_{0}+{\hat{H}}^{*}\;.$$

The interaction term could be represented as ${\hat{H}}^{*}=-e\;\hat{x}\;E\left(t\right)$, where E(t) corresponds to the total field composed of the sum of the two fields $E_{c}\left(t\right)$ and $E_{p}\left(t\right)$, as mentioned above. In a semi-classical approximation, these fields were represented as $E_{c}\left(t\right)=E_{c}\;\cos\left(\omega_{c}t\right)$ and $E_{p}\left(t\right)=E_{p}\;\cos\left(\omega_{p}t\right)$ for the control laser and probe, respectively. At this point, it was optimal to calculate the dipole matrix elements for *x*-polarized incident radiation, $M_{01}=\langle \Psi_{0}\left|e\;x\right|\Psi_{1}\rangle$, $M_{12}=\langle \Psi_{1}\left|e\;x\right|\Psi_{2}\rangle$, and $M_{02}=\langle \Psi_{0}\left|e\;x\right|\Psi_{2}\rangle$. It should be noted that in this development, the condition $M_{02}<<M_{01},M_{12}$ had been assumed. Using Equation (18) and implementing the formalism of the time evolution of the density matrix, we obtained an expression for the system susceptibility $\chi \left(\omega_{p}\right)=\chi_{R}\left(\omega_{p}\right)+i\chi_{I}\left(\omega_{p}\right)$, where the real and imaginary parts, $\chi_{R}\left(\omega_{p}\right)$ and $\chi_{I}\left(\omega_{p}\right)$, respectively, were obtained by: [36]

(19) $$\chi_{R}\left(\omega_{p}\right)=\frac{\Xi \;\Delta (\omega_{p})}{\Theta (\omega_{p})}\left(\frac{|\Omega_{c}{|}^{2}}{4}-\gamma_{02}^{2}-\Delta {\left(\omega_{p}\right)}^{2}\right)\;$$

and

(20) $$\chi_{I}\left(\omega_{p}\right)=\frac{\Xi }{\Theta (\omega_{p})}\left(\gamma_{02}\left(\frac{|\Omega_{c}{|}^{2}}{4}+\gamma_{01}\gamma_{02}\right)+\Delta {\left(\omega_{p}\right)}^{2}\gamma_{01}\right)\;,$$

where $\Xi =|M_{01}{|}^{2}/\epsilon_{0}\hbar$, $\Theta \left(\omega_{p}\right)={\left[\left(\right|\Omega_{c}{|}^{2}/4)+\gamma_{01}\gamma_{02}-\Delta {\left(\omega_{p}\right)}^{2}\right]}^{2}+\Delta {\left(\omega_{p}\right)}^{2}{\left(\gamma_{01}+\gamma_{02}\right)}^{2}$, and $\gamma_{ij}$ are the decay rates and are related to the natural decay rates of the i,j states; and $\Delta \left(\omega_{p}\right)=\left[\left(E_{1}-E_{0}\right)-\hbar \omega_{p}\right]/\hbar$ is the detuning of the system with the probe field. However, $\Omega_{c}=E_{c}\;\left|M_{12}\right|/\hbar$ and $\Omega_{p}=E_{p}\;\left|M_{01}\right|/\hbar$ were the complex Rabi frequencies associated with each laser. By using the intensity of the electromagnetic field associated with a wave of amplitude $E_{c}$ (control field) $I_{c}=c\;\epsilon_{0}{\left|E_{c}\right|}^{2}/2$, an expression was obtained for the intensity of the electromagnetic wave as a function of the associated Rabi frequency and the dipole element $|M_{12}|$ that coupled the states $\Psi_{1}$ and $\Psi_{2}$:

(21) $$I_{c}=\frac{c\;\epsilon_{0}\;\hbar^{2}}{2}\;\frac{|\Omega_{c}{|}^{2}}{|M_{12}{|}^{2}}\;,$$

In this work, the value of the Rabi frequency $\Omega_{c}$ was fixed [37], which implied that the intensity of the electromagnetic radiation (control field) was inversely proportional to the square of the dipole element. An equivalent expression to (21) was obtained for the probe field intensity by using $|\Omega_{p}|$ and $|M_{01}|$.

From Equation (20), an expression for the system absorption was obtained,

(22) $$\alpha_{EIT}\left(\omega_{p}\right)=\left(\omega_{p}/c\right)\;\chi_{I}\left(\omega_{p}\right)\;,$$

where *c* is the speed of light in a vacuum. All the parameters associated with the EIT calculation are included in the following section.

## 3. Results and Discussion

Table 1 details the parameters used throughout the work. Furthermore, $\gamma_{01}$ and $\gamma_{02}$ parameters, as corresponded to the decay rates, were based on the GaAs and AlGaAs materials, in reference to the work of E. B. Al et al. [37] and D. Bejan et al. [38]. Moreover, the $\tau_{in}$ relaxation time was referenced from the reports of E.B. Al et al. [39] and H. Dakhlaoui et al. [13], approximating a constant value since the range of variation of donor density was relatively small. The doping levels that were used for the calculation were in a high doping regime, but their order of magnitude was not a novelty for investigations of this type of heterostructures, both experimentally and theoretically, as evidenced by Y. A. Aleshchenko et al. [40], H. Dakhlaoui [24], and R. B. Dhafera et al. [41].

It should be noted that in this work, the same effective mass was assumed for the well and barrier regions, and the dielectric constant. These approximations did not modify the physics of the results, which was one of the main objectives of the analysis in this work. Furthermore, for the GaAs/AlGaAs heterostructures with 30% Al, the difference in effective masses and dielectric constants did not definitively modify the system’s energies.

**Figure 5:**
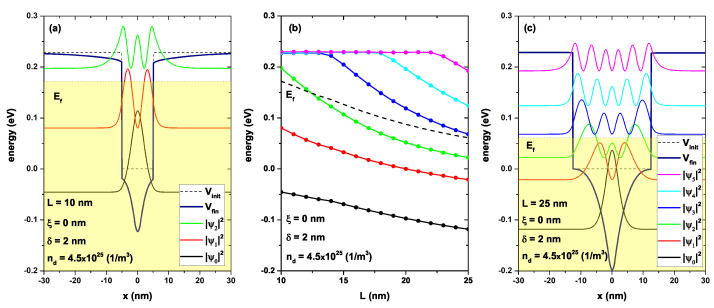
(**a**,**c**): Self-consistent potential V(x) with a solid line in navy color, and electron confinement potential $V_{c}\left(x\right)$ as a black dashed line. Figure (**a**) corresponds to L=10 nm and Figure (**c**) to L=25 nm; for both figures, the parameters ξ=0, $n_{d}=4.5$ × 10${}^{25}$ (1/m${}^{3}$), and δ=2 nm were set. In both figures, the probability densities of all confined states were included. The shaded region indicates the occupied states, and the maximum of this region corresponded to the energy Fermi level in the system. Figure (**b**) represents the lowest confined energy levels as a function of the well width; the dashed line is the self-consistent Fermi level $E_{f}$.

Figure 5 depicts the lowest energy levels for a confined electron in a GaAs/AlGaAs as a function of the well width, considering a delta layer at the well center, Figure 5b. Figure 5a,c show the confinement potential and the probability density for L=10 nm and L=25 nm, respectively. Figure 5a shows the profile of the bottom of the conduction band without including the donor layer (dashed black curve) for L=10 nm, the on-center donor layer with δ = 2 nm, and the donor density $n_{d}$ = $4.5\times 10^{25}$ (1/m${}^{3}$). This profile was modified (continuous navy color curve), causing a sharp profile in the center of the well and a systematic decrease in the barrier regions. The shaded region corresponded to the system-occupied states. The self-consistent Fermi level used an approximate value of 0.175 eV. Additionally, the probability densities associated with the first three confined states were presented (black, red, and green curves). Note that only the $\Psi_{0}$ and $\Psi_{1}$ states were occupied. It was evident that by including the effect of the doped delta layer, a redshift was induced in all states due to the decrease in the value at the bottom of the conduction band. Figure 5c is equivalent to Figure 5a, except for L=25 nm. Note that with this increase in the well width, the number of confined states increased, which was expected since the confinement was decreased. However, there was a decrease in the Fermi level at approximately 0.065 eV, and this presented an occupation of three states, instead of two, as occurred when L=10 nm. For the greater value of the well width, there were no significant modifications in the barrier region (AlGaAs). This was due to the location of the delta layer in the central region of the well and the condition of zero electrostatic potential at infinities; that is, the band offset potential was much greater than the electrostatic potential in these regions. A different situation occurred in the quantum-well region, in which the electrostatic potential significantly modified the bottom of the well, accommodating the lowest states in the this region (see the black and red curves in Figure 5c). Note that the electronic probability was concentrated almost entirely inside the well generated by the delta layer. Figure 5b shows the energy spectrum as a function of the well width while the other parameters are fixed: ξ=0, δ=2 nm, and $n_{d}=4.5\times 10^{25}$ (1/m${}^{3}$). A monotonic decrease was evident for all the confined states (states inside the well). This was due to a decrease in the confinement caused by the increase in the well width. The dashed black line represented the Fermi-level behavior with the increase in *L*. It was observed that the system with L=10 nm presented only two occupied states, as previously mentioned. For a well width greater than 13 nm, the system already presented 3 occupied states. Note the difference in energy between the states became smaller with the increase in *L*. This behavior was due to the decrease in confinement allowing the entry of new eigenvalues from the continuum towards the interior of the quantum well, causing the energies to become closer.

**Figure 6:**
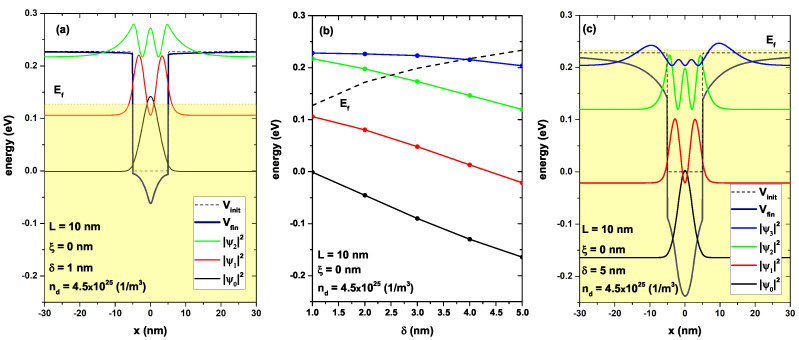
(**a**,**c**): Self-consistent potentials V(x), with a solid line in navy color, and electron confinement potential $V_{c}\left(x\right)$ in black dashed line. Figure (**a**) corresponds to δ=1 nm and Figure (**c**) to δ=5 nm. For both figures, the parameters L=10 nm, ξ=0, and $n_{d}=4.5$ × 10${}^{25}$ (1/m${}^{3}$) were set. In both figures, the probability densities of all confined states were included. The shaded region indicates the occupied states, and the maximum of this region corresponded to the energy Fermi level. Figure (**b**) represents the energy of the confined states according to the δ-parameter; the dashed line is the self-consistent Fermi level $E_{f}$.

In Figure 6, it depicts the lowest energy levels for a confined electron in a GaAs/AlGaAs as a function of the on-center δ-parameter, Figure 6b. Figure 5a,c show the confinement potential and the probability density for δ=1 nm and δ=5 nm, respectively. Figure 6a shows the profile of the conduction band bottom for a quantum well of width *L* = 10 nm, not including the doped delta layer (black dashed curve). This profile corresponded to the electron confinement potential $V_{c}\left(x\right)$ before starting the self-consistent process required to solve the system, including a donor density. The potential profile after self-consistency of the same quantum well, including a doped delta layer in the center (ξ=0), is shown in navy color, width δ=1 nm and $n_{d}=4.5\times 10^{25}$ (1/m${}^{3}$). The shaded region represents the occupied states, and the curves in black, red, and green colors represent the probability density associated with the lowest three confined states, respectively. Figure 6c shows the same system but with δ=5 nm; that is, the region in which the donor density had been distributed was increased. Therefore, for a region of 5 nm, it was not rational to discuss a delta-type doping; the whole region had been doped. The most evident effect was observed in the depth of the quantum well, which now presented the bottom at approximately −0.24 eV, as the energy zero remained fixed at the bottom of the electron confinement potential; there was a clear decrease in the system energy, as compared to the system of δ=1 nm. This modification in the bottom of the conduction band induced the appearance of new confined states, as shown in Figure 6c, the $\Psi_{3}$ state (blue color curve). However, since the volume of the doped layer had been increased with a fixed donor density, this implied that the number of charge carriers must increase, and therefore, the Fermi level in the system must also increase. This was evidenced in the shaded region that now had a much higher value, even above the bottom of the band in the barrier region, causing a full occupation of all confined states. Figure 6b shows the energy spectrum corresponding to the confined states in the quantum well as a function of the width of the δ-parameter, with fixed L=10 nm, ξ=0, and $n_{d}=4.5\times 10^{25}$ (1/m${}^{3}$). The dashed line corresponds to the system’s Fermi level that presented a monotonically increasing behavior, as previously mentioned. All the states presented a redshift in energy, and the shift was more abrupt for lower states; for example, the ground state presented a shift of approximately 0.16 eV. This decrease in energies, despite having a clear increase in confinement with the increase in the δ-parameter, was because the energy had been measured according to the bottom of the electron confinement potential $V_{c}\left(x\right)$ (dashed line in Figure 6a) and not for the lowest point of the conduction band that corresponded to the center of the doped layer.

**Figure 7:**
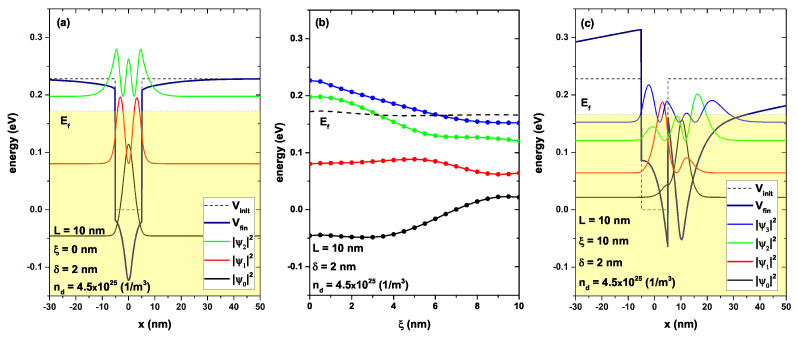
(**a**,**c**): Self-consistent potentials V(x) with a solid line in navy color, and electron confinement potential $V_{c}\left(x\right)$ in black dashed line. Figure (**a**) corresponds to ξ=0 and Figure (**c**) to ξ=10 nm. For both figures, the parameters L=10 nm, $n_{d}=4.5$ × 10${}^{25}$ (1/m${}^{3}$), and δ=2 nm were set. In both figures, the probability densities of some confined states were included. The shaded region indicates the occupied states and the maximum of this region corresponded to the energy Fermi level. Figure (**b**) represents the lowest confined energy levels as a function of the ξ-parameter; the dashed line is the self-consistent Fermi level $E_{f}$.

In Figure 7, the lowest energy levels are depicted for a confined electron in a GaAs/AlGaAs as a function of the on-center ξ-parameter in Figure 7b. Figure 7a,c show the confinement potential and the probability density for ξ=0 and ξ=10 nm, respectively. Figure 7a is the same as Figure 5a, but with a different scale for comparison purposes with Figure 7c. This showed the quantum well with delta doping remained fixed L=10 nm, ξ=0, and $n_{d}=4.5$ × 10${}^{25}$ (1/m${}^{3}$). Figure 7c shows the same system, but the doped delta layer was located at ξ=10 nm, that is, it was 5 nm outside the edge of the well. The movement of the delta layer to the right caused the symmetry of the system to be lost; a direct implication of this was that the wave functions could also lose symmetry. In this case, an accumulation of charge carriers was generated in the region where the delta layer had been located, causing an electrostatic potential with a significantly high value at that point (as compared to the confinement potential $V_{0}$). As a consequence, the total self-consistent potential (the sum of the confinement potential and the Hartee potential) was generated for a double-well system, as shown in Figure 7c. The AlGaAs barrier region had a clear asymmetry in the potential, including $V\left(x\right)>V_{0}$ for x<−5 nm and $V\left(x\right)<V_{0}$ for x>10 nm; this difference in the electronic confinement caused the probability density associated with the ground state to accumulate to a greater extent in the well produced by the delta layer (the electrons tended to be located in the place of least confinement), around x=10 nm. Note the opposite occurred with the first excited state, which remained at high density inside the GaAs well in −5 nm <x<5 nm.

However, from a practical perspective, the Fermi level did not change with the displacement of the delta layer; this was because the change in ξ did not modify the number of charge carriers. However, this did not imply that the number of occupied states could be modified; as shown in Figure 7a, there were two occupied states while in Figure 7c, there were already four occupied states. Locating the delta layer in a position that broke the symmetry of the system also generated a break in the symmetry of the self-consistent potential (V(x)). This was mainly caused by the electrostatic potential that now presented a high asymmetry, drastically modifying the electron confinement potential of the well. In Figure 7b, we observed the energies of the first four confined states as a function of the position of the ξ-parameter, while the others parameters remained fixed at L=10 nm, δ=2 nm, and $n_{d}=4.5$ × 10${}^{25}$ (1/m${}^{3}$). The Fermi level corresponded to the dashed line, and this remained practically fixed, as previously stated, since there was no change in the number of carriers or in the temperature of the system. Note that the ground state and the first excited state (black and red curves, respectively) remained approximately unchanged in the range 0≤ξ<5 nm; this was the range in which the delta layer had moved from the center to the edge of the quantum well, and there was no change in the depth of the well. In the range ξ>5 nm, these two states had demonstrated a tendency to come together; this was because the delta layer induced a well width of around 35 nm, resulting in the origination of a new quantum well, and the system behaved as a coupled double-quantum-well system.

**Figure 8:**
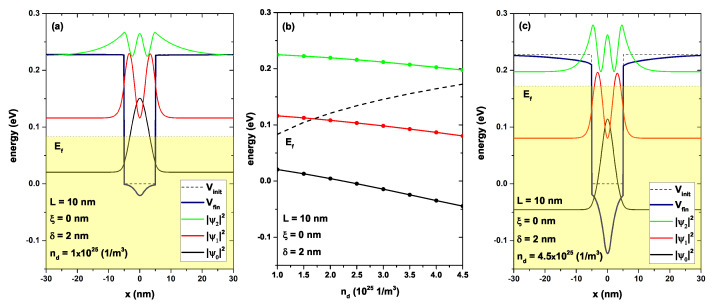
(**a**,**c**): Self-consistent potentials V(x) with a solid line in navy color, and electron confinement potential $V_{c}\left(x\right)$ in black dashed line. Figure (**a**) corresponds to $n_{d}=1.0$ × 10${}^{25}$ (1/m${}^{3}$) and figure (**c**) to $n_{d}=4.5$ × 10${}^{25}$ (1/m${}^{3}$). For both figures, the parameters L=10 nm, ξ=0 nm, and δ=2 nm were set. In both figures, the probability densities of some confined states were included. The shaded region indicates the occupied states, and the maximum of this region corresponded to the energy Fermi level. Figure (**b**) represents the energy of the confined states as a function of $n_{d}$-parameter; the dashed line is the self-consistent Fermi level $E_{f}$.

In Figure 8, the lowest energy levels are shown for a confined electron in a GaAs/AlGaAs as a function of the on-center $n_{d}$-parameter, Figure 8b. Figure 8a,c show the confinement potential and the probability density for $n_{d}=1.0$ × 10${}^{25}$ (1/m${}^{3}$) and $n_{d}=4.5$ × 10${}^{25}$ (1/m${}^{3}$). Figure 8a shows the quantum well system with L=10 nm, ξ=0, δ=2 nm, and $n_{d}=1.0$ × 10${}^{25}$ (1/m${}^{3}$). As the donor density was lower, there was no significant change in the barrier regions and, in the well, only a decrease of approximately 0.06 eV. The Fermi level had to change when the donor density decreased; in this case, it had a value of 0.13 eV (shaded region) and corresponded to the occupation of the ground state (see the black curve in Figure 8a). Figure 8c shows the same system but now with an increase in the donor density to $n_{d}=4.5$ × 10${}^{25}$ (1/m${}^{3}$), while the other parameters remained the same. Firstly, it was evident that the increase in donor density generated a deeper quantum well inside the initial well (compare $V_{c}\left(x\right)$ with V(x)). However, the states presented a slight decrease concerning zero energy, and the probability density tended to be a little more localized around the point x=0. In Figure 8b, the energy levels are presented as a function of the donor density $n_{d}$ for the first three states, fixing the width of the well, the position of the delta layer, and the width of the delta layer. The Fermi level again corresponded to the dashed line and presented a monotonically increasing behavior, which was a result of the increase in the number of charge carriers of $n_{d}$. For the lowest density, that is, for $n_{d}=1.0$ × 10${}^{25}$ (1/m${}^{3}$), only the ground state was occupied; for $n_{d}>1.75$×$10^{25}$ (1/m${}^{3}$), there began to be an occupation of the first excited state. As the donor density increased, the bottom of the well began to decrease towards lower energies, as shown in all confined states. However, it should be noted that the relative distance between the bottom of the conduction band (bottom of the quantum well) and each one of the states presented an increase that had been originated by the increase in confinement; that is, the electrons experienced a greater height well with an increase in $n_{d}$.

**Figure 9:**
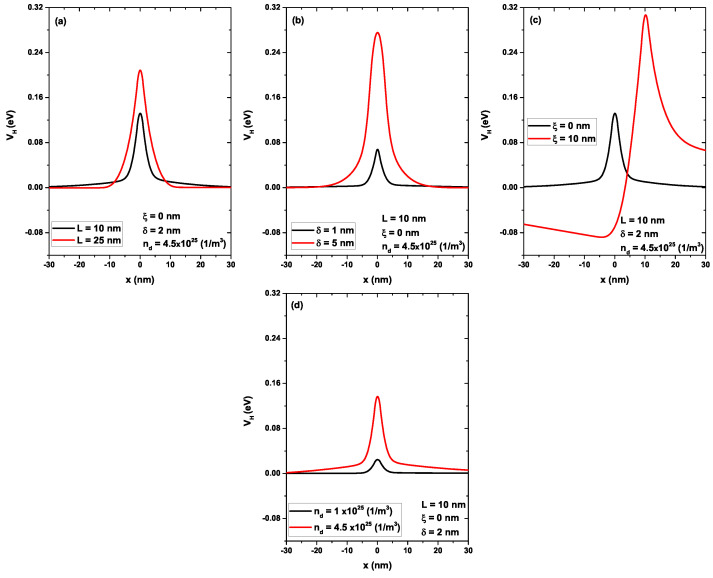
Self-consistent Hartree potential as a function of *x*-position. In (**a**) varying the *L*, in (**b**) varying the δ, in (**c**) varying the ξ, and in (**d**) varying the $n_{d}$. In each case, when one parameter was changed, the others were set to L=10 nm, ξ=0, δ=2 nm, and $n_{d}=4.5$ × 10${}^{25}$ (1/m${}^{3}$).

Figure 9 shows the self-consistent Hartree potential as a function of position *x*: in (a) varying the *L*, in (b) varying the δ, in (c) varying the ξ, and in (d) varying the $n_{d}$. In each case, when one parameter changed, the others were set to L=10 nm, ξ=0, δ=2 nm, and $n_{d}=4.5$ × 10${}^{25}$ (1/m${}^{3}$). Figure 9a–d were reviewed at the same scale for comparison purposes. Note that the Hartree potential with the smallest value was the one corresponding to $n_{d}=1.0$ × 10${}^{25}$ (1/m${}^{3}$) (black curve in Figure 9d); that is, with this density value of the delta layer, there were no major modifications in terms of the bottom profile of the conduction band since it had a value comparable to the electron confinement potential ($V_{0}$); this was previously shown in Figure 8a by comparing the potentials $V_{c}\left(x\right)$ and V(x). The increase in the density of the donors generated an increase in the electrostatic potential, as shown in the red curve of Figure 9c. Figure 9b shows the significant increase in the Hartree potential due to the increase in the doped region, from δ=1 nm to δ=5 nm (black and red curves, respectively). This modification explained the increase in the depth of the quantum well in Figure 6c and the change in the shape of the barrier region because the potential adopted values, as compared to the barrier potential $V_{c}$, for points in the regions x<−5 nm and x>5 nm (see the red curve in Figure 9b). However, Figure 9a shows an increase in the electrostatic potential caused by the increase in the well width, from L=10 nm to L=25 nm. Although there was no change in the donor density, increasing the well width generated a much wider region in which the electrons could be distributed, changing the shape of the electron density (which is shown later) and, therefore, the Fermi level, as well as the occupied states, as previously presented in Figure 5c. Finally, in Figure 9c, by comparing the black curve that corresponds to ξ=0 with the red curve for ξ=10 nm, the asymmetry that had originated in the Hartree potential by the position of the delta layer that had induced an asymmetry initiated a second potential well at the bottom of the conduction band, as previously presented in Figure 7c with asymmetric states.

**Figure 10:**
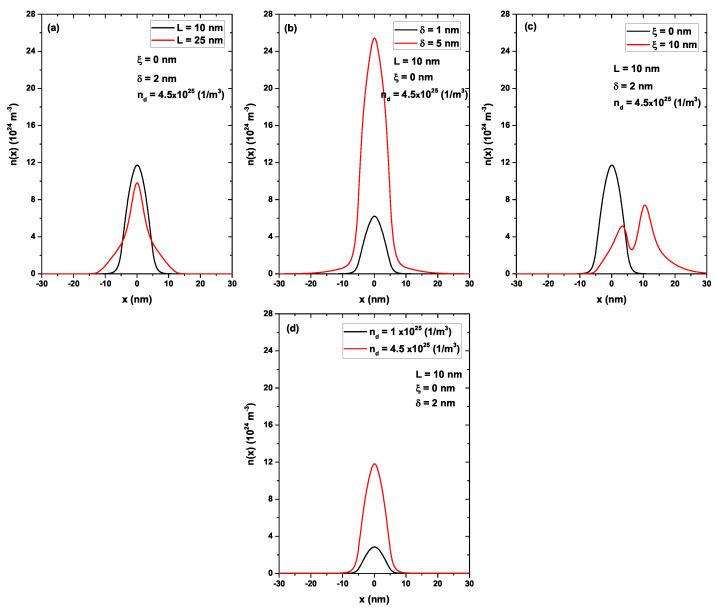
Electron density as a function of *x*-position. In (**a**) varying the *L*, in (**b**) varying the δ, in (**c**) varying the ξ, and in (**d**) varying the $n_{d}$. In each case, when one parameter was changed, the others were set to L=10 nm, ξ=0, δ=2 nm, and $n_{d}=4.5$ × 10${}^{25}$ (1/m${}^{3}$).

Figure 10 shows the electron density as a function of the *x*-position: in (a) varying the *L*, in (b) varying the δ, in (c) varying the ξ, and in (d) varying the $n_{d}$. In each case, when one parameter was changed, the others were set to L=10 nm, ξ=0, δ=2 nm, and $n_{d}=4.5$ × 10${}^{25}$ (1/m${}^{3}$). Again, all four figures have been compared according to on the same scale. In Figure 10a, the electron density was compared for two different widths of the quantum well. Increasing *L* did not generate changes in the donor density; therefore, in magnitude, the density had remain fixed, as only its distribution changed. Therefore, for L=10 nm, the electrons were more localized in the central region between −5 nm <x< 5 nm. However, for L=25 nm, the electrons lost the location and were now located in a much larger region, between −12.5 nm <x< 12.5 nm, while the number of electrons per unit volume remained fixed. Figure 10b compares the electron density for two widths of the doped layer δ=1 nm and δ=5 nm. Since there was no change in the well width, the electrons were distributed mainly in the region between −5 nm <x< 5 nm. The electron density magnitude was greater for δ=5 nm since the donor density in $n_{d}=4.5$ × 10${}^{25}$ (1/m${}^{3}$), and these are distributed in a greater volume. Therefore, the number of charge carriers also had to increase, as clearly evidenced by comparing the red and black curves. Figure 10c compares the electron density for two different positions of the same doped delta layer: ξ=0 and ξ=10 nm, located 5 nm to the right of the edge of the well. For ξ=0, once again, the electrons were distributed inside the GaAs well, while for ξ=10 nm, most of the electrons were already in the AlGaAs region located in the well that had been generated by the doped delta layer. Although a portion of the electrons remained evenly balanced inside the initial well, this distribution was due to the well generated by the delta layer having a slightly greater width than the initial well that it had been modified by the Hartree potential; since this one was sharper, the electrons tended towards the region of least confinement (see Figure 7c). Finally, Figure 10d compares the electron density for two different donor densities in the delta layer $n_{d}=1.0$ × 10${}^{25}$ (1/m${}^{3}$) and $n_{d}=4.5$ × 10${}^{25}$ (1/m${}^{3}$), corresponding to the black and red curves, respectively. In this case, the increase in $n_{d}$ directly caused an increase in the number of negative charge carriers that were concentrated inside the quantum well, which was only modified in its depth.

**Figure 11:**
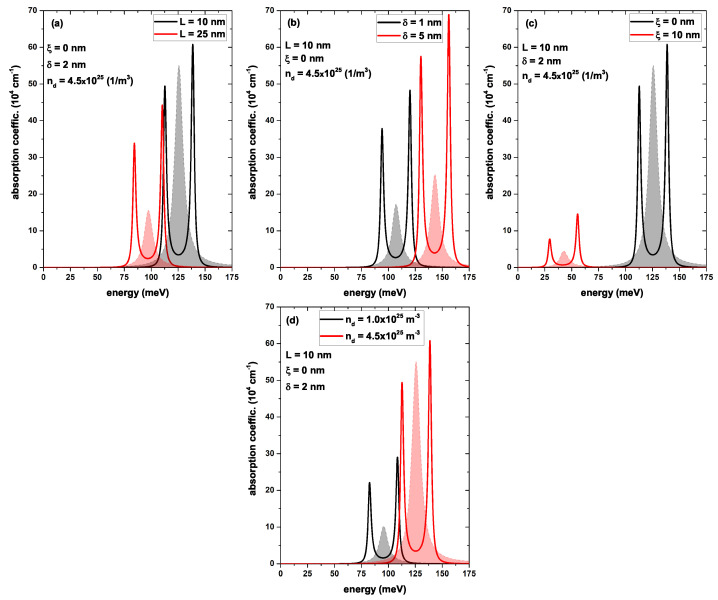
In each figure, the shaded curves represent the absorption coefficient calculated according to Equation (16); between the states $\Psi_{0}$ and $\Psi_{1}$ (the absorption curves have been multiplied by 5), the un-shaded curves correspond to the calculation of the EIT between the first three states of the system calculated using Equation (22). In (**a**) varying the *L*, in (**b**) varying the δ, in (**c**) varying the ξ, and in (**d**) varying the $n_{d}$. In each case, when one parameter was changed, the others were set to L=10 nm, ξ=0 nm, δ=2 nm, and $n_{d}=4.5$ × 10${}^{25}$ (1/m${}^{3}$).

Figure 11 shows the absorption coefficient calculated according to Equation (16) between the states $\Psi_{0}$ and $\Psi_{1}$ (shaded curves. The absorption curves were multiplied by 5) as a function of the incident photon energy. The un-shaded curves corresponded to the calculation of the EIT between the first three states of the system calculated via Equation (22): in (a) varying the *L*, in (b) varying the δ, in (c) varying the ξ, and in (d) varying the $n_{d}$. In each case, when one parameter was changed, the others were set to L=10 nm, ξ=0 δ=2 nm, and $n_{d}=4.5$ × 10${}^{25}$ (1/m${}^{3}$). In Figure 11a, we found that the absorption coefficient corresponding to the system with L=10 nm had a greater intensity than the one corresponding to L=25 nm; this was due to the matrix element $|M_{01}{|}^{2}$ corresponding to the system with the smallest *L* being higher. However, the photon absorption occurred at a higher energy of 125.6 meV for L=10 nm, as compared to the 97.3 meV of L=25 nm. This was expected since the states for a smaller *L* were further apart. As the *L* increased, more states entered the system, and the confined states began to converge, as shown in Figure 5. In Figure 11b, we observed that the peak of the absorption coefficient for δ=1 nm occurred at 107.1 meV, while for δ=5 nm, it occurred at 143.2 meV. This was due to increasing the width of the doped layer, as this increased the Hartree potential and, therefore, the depth of the quantum well, causing the entry of new states and a reduction in the separation of the already confined states. This behavior was evidenced in Figure 6. In Figure 11c, we observed that the maximum peak of the absorption coefficient corresponded to a system with ξ=0, for an energy of 125.5 meV, as compared to the 42.7 meV of the delta layer located in the asymmetric system ξ=10 nm. The explanation for this behavior was evident from the analysis of Figure 7, in which the states $\Psi_{0}$ and $\Psi_{1}$ were closer when the delta layer was in the center of the quantum well. Whereas when the delta layer increased to ξ=10 nm, a double quantum-well system was generated, resulting in the appearance of new confined states and a decrease in the transition energy between the ground state and the first excited state (see the black and red curves of Figure 7b). Finally, in Figure 11d, the absorption peak was 95.6 meV for the system with $n_{d}=1.0$ × 10${}^{25}$ (1/m${}^{3}$) and of a significantly smaller magnitude than for $n_{d}=4.5$ × 10${}^{25}$ (1/m${}^{3}$), which had a value of 125.7 meV. This behavior could be understood through Figure 9c, which showed that the increase in the donor density caused an increase in the Hartree potential, and as a consequence, a deeper quantum well was generated (see Figure 8), causing a separation of the confined states and, particularly, an increase in the transition energy corresponding to $\Psi_{0}\to \Psi_{1}$. The maximum absorption energy was presented for the set of parameters L=10 nm, ξ=0, δ=5 nm, and $n_{d}=4.5$ × 10${}^{25}$ (1/m${}^{3}$), with an energy of 143.2 meV represented by the shaded red curve in Figure 11b.

The EIT was calculated according to the absorption coefficient, which, as stated in the theoretical framework, required three confined states. In this calculation, the ground state and the first two excited states were assigned as $\Psi_{0}$, $\Psi_{1}$, and $\Psi_{2}$, respectively, in a cascade configuration for each of the configurations presented in Figure 11. The emergence of the EIT effect required a coupling between the states $\Psi_{0}-\Psi_{1}$ and $\Psi_{1}-\Psi_{2}$, the Rabi frequency associated with the control field $\Omega_{c}$, and the natural decay rates of the states $\gamma_{01}$ and $\gamma_{02}$ (see Table 1). These decay rates were of great importance in this effect, as they governed the magnitude of the resonant structures. Note that in each graph of Figure 11a–d, when the control field was activated (through the frequency $\omega_{p}$ between states $\Psi_{1}$–$\Psi_{2}$), the points of maximum absorption now changed, becoming points of minimum absorption (see red and black un-shaded curves in Figure 11a–d). Therefore, the system had become almost transparent for the frequency at which its absorption had reached the maximum due to the coupling of a third state with an external control field.

## 4. Conclusions

The effects of a doped layer on the optical absorption coefficient and the EIT value in a GaAs/AlGaAs QW were studied using a self-consistent coupling formalism between the Schrödinger, Poisson, and charge-neutrality equations. The induced modification to the conduction band due to the electrostatic potential originated by the charge carriers was considered. The modifications in the well width increased the number of confined electron states, which generated modifications in the electronic density and, therefore, in the linear absorption coefficient. Consequently, an appreciable decrease in the intensity of the absorption peak and a redshift in the resonance energy were observed; a similar behavior was evident in the EIT. It was shown that by increasing the doped-layer width, a deeper QW was generated, and as a consequence of this, the confined-state occupation was significantly increased. A similar effect was observed in the transition energy between them. An immediate consequence was an increase in the electron density, the electrostatic potential, and a blue shift with higher intensity absorption peaks. However, when the position of the doped delta layer was modified while maintaining a fixed width of 2 nm and a 3-dimensional doping density at $n_{d}=4.5$ × 10${}^{25}$ (1/m${}^{3}$), the self-consistent potential profile became completely asymmetric and resulted in a double-well system. These modifications increased the number of confined states simultaneously, alongside a decrease in transition energies. The direct consequences on the optical properties of the system were observed: the redshift of the peak position and a significant decrease in the intensity of both the linear absorption coefficient and the EIT. To finalize the system characterization, modifications were made in the donor density, causing an increase in the electrostatic potential, the electronic density, and the number of occupied states. In this case, the transition energy between the states was higher, and therefore, there was a blueshift of the resonance peak in the linear absorption and a clear increase in its intensity. In this work, the possibility of tuning the optical properties, such as the linear absorption coefficient and the EIT, was demonstrated using modifications in various geometric and non-geometric system parameters, including the donor density. Our findings demonstrated the potentially useful applications of doped QWs in electronics, science, and engineering.

## Figures and Tables

**Table 1:** List of parameters used in the calculations.

Parameter	Value
*ℏ*	1.054 × 10${}^{-34}$ (J s)
$m^{*}$	0.067 $m_{0}$ (Kg)
$m_{0}$	9.109 × 10${}^{-31}$ (Kg)
*e*	1.602 × 10${}^{-19}$ (C)
$k_{B}$	1.381 × 10${}^{-23}$ (J/K)
$\epsilon_{0}$	8.854 × 10${}^{-12}$ (F/m)
ϵ	12.35
$V_{0}$	0.228 (eV)
*T*	300 (K)
*c*	299,792.458 (m/s)
$\gamma_{01}$	0.1 (THz)
$\gamma_{02}$	5 (THz)
$\Omega_{c}$	40 (THz)
$\tau_{in}$	0.14 (ps)
μ	4π × 10${}^{-7}$ (H/m)

## Data Availability

No new data were created or analyzed in this study. Data-sharing does not apply to this article.
